# Diagnosis, Treatment, and Unmet Needs of Dedifferentiated Liposarcoma in the United States: A Multidisciplinary Delphi Study

**DOI:** 10.3390/cancers17172815

**Published:** 2025-08-28

**Authors:** David Campbell, Scott Ramsey, David Veenstra, Minggui Pan, Shiraj Sen, Gregory Litton, Bruce Brockstein, Shawn Young, Andrew Fang, Parth Shah

**Affiliations:** 1 Curta, Inc., Seattle, WA 98104, USA; scott.ramsey@curta.com (S.R.); david.veenstra@curta.com (D.V.); 2 University of Washington, Seattle, WA 98195, USA; 3 Sarcoma Program, Division of Oncology, Stanford University School of Medicine, Stanford, CA 94305, USA; minggui@stanford.edu; 4 Next Oncology, San Antonio, TX 78229, USA; ssen@nextoncology.com; 5 Intermountain Medical Center, Murray, UT 84107, USA; glitton@utahcancer.com; 6 Endeavor Health, Evanston, IL 60201, USA; bbrockstein@northshore.org; 7 Saint Joseph Hospital, SurgOne, Denver, CO 80218, USA; shawn.young@sclhealth.org; 8 Kaiser South San Francisco Medical Center, San Francisco, CA 94080, USA; andrew.fang@kp.org; 9 Saint Joseph Hospital, Intermountain Health, Denver, CO 80218, USA; parth.shah@imail2.org

**Keywords:** Delphi study, dedifferentiated liposarcoma (DDLPS), unmet need, treatment patterns

## Abstract

**Background**: Evidence of the real-world management of dedifferentiated liposarcoma (DDLPS) is limited by the patient size and coding. The objective of this study is to generate consensus expert opinion on locally advanced or metastatic DDLPS diagnosis, treatment, and unmet needs. **Methods**: A three-round Delphi consensus panel was conducted with 9 DDLPS clinical experts from November to December 2023. Expert panelists were recruited across academic specialty and traditional settings and US regions. The Delphi panel included two rounds of surveys followed by a consensus building workshop. Surveys contained multiple-choice and free response questions, and statements for level of agreement rating. Panelists rated each statement for level of agreement on a 9-point Likert scale. Statements with ≥75% of scores ≥ 7 achieved consensus, and those that did not achieve consensus agreement were modified or removed from subsequent testing. A virtual workshop was held to discuss areas which did not achieve consensus and refine previously agreed upon statements. **Results**: In total 25 consensus statements were developed by the Delphi panel. Survey 1 achieved 7 consensus statements across the areas of burden, treatment, and unmet needs of DDLPS. Survey 2 generated an additional 10 consensus statements. During the workshop, eight more statements achieved consensus, and four statements were refined for enhanced clarity and precision. The study findings are limited by the number of Delphi panel participants and consensus statements may not be fully representative of clinician perspectives across the US. **Conclusions**: Consensus areas identified by the Delphi panel help better understand the decision factors for surgical and non-surgical treatments and anticipated utilization. These results could be used to inform both drug development programs as well as care delivery challenges for liposarcoma patients.

## Simple Summary

Dedifferentiated liposarcoma (DDLPS) is a rare disease with limited treatment options and poor prognosis. A Delphi study with clinical experts across the United States convened to generate consensus statements on the diagnosis, treatment, and unmet needs of DDLPS. Panelists reached agreement on the preferred first-line and subsequent treatments currently available, and emphasized the need for novel treatments with improved efficacy and safety. The insights generated by the Delphi panel improve the understanding of clinical decision factors for surgical and non-surgical treatments and anticipated utilization of novel therapies.

## 1. Introduction

Liposarcoma accounts for approximately 15% to 20% of all soft tissue sarcomas [1]. There are several subtypes of liposarcoma, including well-differentiated liposarcoma (WDLPS), dedifferentiated liposarcoma (DDLPS), pleomorphic liposarcoma and myxoid liposarcoma. DDLPS can occur as a de novo malignancy or transform from WDLPS [2]. There are less than 1000 new cases of DDLPS per year in the United States; however, patients with DDLPS have a poor prognosis with an estimated survival rate of 50% at 5 years, and 33% at 10 years [3,4,5]. For localized DDLPS after surgical resection, local recurrence rates are high at 40–60%, while distant metastases occur in 15–30% of cases [1,6].

DDLPS is characterized by gene amplification of MDM2 gene in nearly all cases. In addition, the majority of DDLPS also possess amplification of CDK4. These gene amplifications have been thought to be key drivers of DDLPS oncogenesis [7,8]. Amplification of MDM2 results in overexpression of MDM2 protein which binds to and induces degradation of p53, a tumor suppressor gene that carries out many critical cellular functions to prevent oncogenesis [9,10,11]. When DDLPS develops in the retroperitoneum, tumors may grow very large in size before signs or symptoms, such as abdominal pain, back pain, bowel obstruction, nausea, early satiety, or a palpable mass are detected [12]. Disease in the extremity will often present as a painful or painless palpable mass [13]. The rarity and variable presentation of soft tissue sarcomas including DDLPS make timely diagnosis challenging [14]. Limited awareness and experience with the management of DDLPS among primary and secondary care physicians can result in multiple referrals to different specialties before diagnosis of a correct liposarcoma subtype is made [15]. Identification of myxoid stroma by pathologists may result in misdiagnosis of DDLPS as myxoid liposarcoma or myxofibrosarcoma [13,16].

Surgery with or without radiotherapy is the standard of care for the majority of resectable DDLPS. Systemic therapy is rarely utilized as first-line treatment when the disease is resectable. DDLPS in an extremity is routinely treated with neoadjuvant radiotherapy followed by surgical resection. However, retroperitoneal DDLPS can be much more challenging to manage due to its large size and location which may invade or be in close apposition to critical structures impeding the ability to achieve R0 resection. DDLPS in the retroperitoneum may or may not be treated with neoadjuvant radiotherapy, and the decision requires multidisciplinary discussion and remains controversial [6]. In the international STRASS study, neoadjuvant radiotherapy for retroperitoneal sarcomas was found to not increase median abdominal recurrence-free survival [17]. The treatment options for locally advanced unresectable or metastatic DDLPS are limited. Doxorubicin has remained a standard first-line treatment but with limited efficacy [8,18,19]. In a recent study, the median overall survival for patients with advanced DDLPS was approximately 15 months [20].

Due to a lack of specific diagnostic codes, and a small number of patients, identification of DDLPS patients for the generation of real-world evidence from claims and electronic health records data is difficult. Subsequently, there is only limited evidence describing the burden and treatment patterns. Delphi studies are a robust and well-established method for consensus building that has been used to reach consensus agreement from diverse stakeholders [21,22]. The aim of the Delphi panel study was to generate consensus expert opinion on DDLPS diagnosis, treatment, and unmet needs to support the development of novel DDLPS therapies and management strategies.

## 2. Materials and Methods

### 2.1. Selection of Panelist

Nine United States-based clinical experts in the diagnosis and management of DDLPS including surgical, musculoskeletal, and medical oncologists were recruited to be Delphi study panelists. Expertise in the management of DDLPS was assessed by years in practice, publications, and participation in sarcoma clinical trials. While panelist size varies across Delphi studies, the size of the panel assembled in this study aligned with similar studies for cancer management [23,24,25]. Panelists were recruited across academic specialty and community settings as well as geographic regions to enhance the generalizability of consensus statements (Table 1).

### 2.2. Study Design

**Figure 1:**
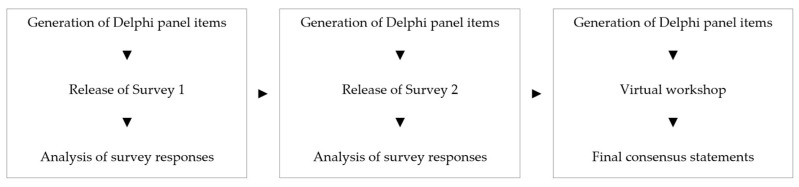
Delphi study process.

Consistent with accepted practice for Delphi studies, the approach consisted of two rounds of surveys followed by a consensus meeting [26,27] (Figure 1). Surveys contained a mix of statements to be rated for level of agreement by panelist and free response questions to elucidate potential areas of consensus. Statements which did not achieve consensus were revised or removed in subsequent polling. Revised statements were discussed, refined, and rated in a consensus gathering virtual workshop.

### 2.3. Data Collection and Analysis

Survey 1 included a mix of statements for level of agreement rating, multiple-choice, and free response questions. Eleven statements for level of agreement rating were selected for inclusion within survey 1, which aimed to address modeling gaps under the domains of diagnosis, treatment, and unmet needs of DDLPS. In addition, seven free response questions, and 12 multiple-choice questions were included to narrow the scope and generate additional statements for consensus rating. Panelists rated each statement on a 9-point Likert scale. Consensus agreement was defined as ≥75% of experts scoring a statement with ≥7. Statements which achieved consensus in survey 1 were excluded from survey 2 and presented at the virtual workshop stage for further refinement. Statements where panelists indicated disagreement with ≥75% of experts scoring the statement as ≤3 were rejected and not included in survey 2. The remaining statements with neutral rating were modified or combined for subsequent testing. Survey 2 contained 12 statements for consensus rating, 2 free response questions, and 4 multiple-choice questions. During the virtual workshop, 9 new statements were rated for level of agreement among panelist, and 17 previously agreed upon statements were reviewed for further clarity.

## 3. Results

Eight study participants completed Survey 1 and Survey 2, and 9 study participants attended the virtual Delphi study workshop on 4 December 2023. By the end of the Delphi workshop, a total of 25 statements achieved consensus agreement across the domains of DDLPS diagnosis, treatment, and unmet needs. Survey 1 contained 30 questions including 7 free response questions, 12 multiple-choice questions, and 11 statements for level of agreement rating, of which 7 statements achieved consensus agreement. Survey 2 contained 18 questions including 2 free response questions, 4 multiple-choice questions, and 12 statements for level of agreement rating of which 10 achieved consensus agreement. Eight additional statements achieved consensus either through the process of revision or through the generation of new statements within discussion. In addition, 4 statements which had previously achieved consensus were refined by the panel for enhanced clarity and precision. The full list of consensus and non-consensus statements from Survey 1, Survey 2, and virtual workshop are available in the Supplementary Materials.

### 3.1. Diagnosis of DDLPS

The Delphi panel reached consensus agreement on five statements to characterize the presentation, timing, and patient journey involved in the diagnosis of DDLPS (Table 2). The panel agreed that the likelihood a patient with DDLPS will present with symptoms at the time of diagnosis varies by primary tumor location (statement 1). Depending on tumor location, symptoms may include pain, palpable mass, or body asymmetry. Many disciplines including surgical oncology, orthopedic oncology, radiology, interventional radiology, and pathology are typically consulted in the pathway to diagnose DDLPS (statement 2). The panel estimated patients will on average have at least 2–3 referrals before a DDLPS diagnosis is made (statement 3). An accurate DDLPS diagnosis is typically made within a year from symptom onset (statement 4). However, the time from symptom onset to diagnosis can be delayed by many factors. Panelists agreed that the common reasons affecting the timely identification, and diagnosis of DDLPS patients include non-specific symptoms, delays in receiving biopsy or imaging, and clinician misdiagnosis (statement 5).

### 3.2. Treatment of DDLPS

The Delphi panel reached consensus agreement on 12 statements for the current and optimal treatment of DDLPS (Table 3). Among patients diagnosed with DDLPS, surgery with or without radiation or chemotherapy is the preferred first treatment in early-stage, localized disease with no or limited spread to nearby lymph nodes. Following diagnosis, approximately 80% of stage 3 DDLPS patients undergo an oncologic surgery as part of a treatment strategy with curative intent (statement 6, statement 7). For patients with stage 3 DDLPS, surgical morbidity, tumor location, expected adequacy of margins, and comorbidities are the most influential factors in determining if a patient should or should not receive surgery as first-line of treatment (statement 8). Among stage 3 patients for whom surgery is not recommended, the panel estimated that approximately 80% will receive systemic therapy (statement 9). Among patients that experience disease progression after receiving surgery as initial treatment, the most important factors for selecting the next treatment are tumor location, resectability, symptom burden, performance status, time interval since surgery, and presence of metastases (statement 10). A systemic therapy is recommended over additional surgery if disease has progressed distantly within 6 months of the surgery (statement 11). For patients initially diagnosed as stage 3 who progress after surgery, approximately 80% will receive systemic therapy (statement 12).

An estimated 30% of stage 4 DDLPS patients receive palliative surgery with utilization dependent on primary tumor location, and approximately 90% will receive systemic therapy during their treatment journey (statement 13, statement 14). Doxorubicin-based chemotherapy is the preferred and most used first-line treatment for unresectable or metastatic DDLPS (statement 15, statement 16). Following initial therapy with doxorubicin, the most used treatment regimens are ifosfamide, gemcitabine with or without docetaxel, pazopanib, trabectedin, eribulin, palbociclib (statement 17). The pivotal trial of pazopanib (PALLETTE) did not include patients with adipocytic tumors; however, other studies have shown that pazopanib does have activity in WDLPS and DDLPS [28,29,30,31].

### 3.3. DDLPS Unmet Need

The Delphi panel reached consensus agreement on 8 statements related to the unmet needs and expected benefits of novel DDLPS treatments (Table 4). For patients with stage 3 or 4 DDLPS, the panelists agreed that the efficacy of available systemic therapies remains poor, and the greatest unmet need is for more efficacious systemic therapies (statement 18). The panel identified a need for a first-line systemic treatment with improved efficacy, safety, and tolerability in the treatment of locally advanced (unresectable) or metastatic DDLPS (statement 19, statement 20). For patients with unresectable DDLPS, the greatest need is for effective systemic treatment that can be used prior to other currently available systemic therapies (statement 21).

The panel emphasized the significant burden of DDLPS extends beyond the patient. Locally advanced (unresectable) or metastatic DDLPS significantly limits patient and caregivers’ daily personal and/or professional life (statement 22). The panel estimated that among patients who receive surgery for DDLPS, between 1 and 4 surgeries are typical before initiating systemic therapy (statement 23). Due to the limitations of current systemic treatment options, patients may receive repeated surgeries. The availability of a new systemic treatment that offers substantial improvement in efficacy over doxorubicin has the potential to reduce the average number of surgeries before the initiation of systemic therapy (statement 24). A new systemic treatment for DDLPS which reduces the need for additional surgeries would meaningfully reduce patient and caregiver burden (statement 25).

## 4. Discussion

This was the first Delphi panel to address evidence gaps related to the diagnosis, treatment, and unmet needs of DDLPS within the United States. Towards this aim, the consensus statements from the expert panel in this study may provide guidance to enhance patient care, overcome treatment challenges, and inform drug development programs by identifying where the greatest needs remain for liposarcoma patients.

The Delphi panel consensus statements on the diagnostic journey of DDLPS highlight the challenges with timely diagnosis for a rare cancer. Experts agreed that the non-specific symptoms, delays in receiving biopsy or imaging, and clinician misdiagnosis are all common reasons affecting the timely identification, and diagnosis of DDLPS. Further, the average patient journey to diagnosis includes multiple clinical referrals and consultations with surgical oncology, orthopedic oncology, radiology, interventional radiology, and pathology specialists. Reducing the time to diagnosis is an important step towards enhancing clinical outcomes. The consensus recommendations in this study recommend pathologists to be included within the multidisciplinary team and patients receive biopsy quickly to avoid diagnostic delays. A recent multidisciplinary position statement goes further and recommends; given the increasing role of histology-specific therapeutic approaches, individuals with a suspected DDLPS should have pathology reviewed at a center with sarcoma expertise, and the use of molecular diagnostics, including the assessment of MDM2 amplification by FISH, or, in the appropriate clinical context, overexpression by IHC, can help confirm a diagnosis of DDLPS [13]. Panelist reached agreement on the preferred first-line and subsequent treatments currently available and emphasized the need for novel treatments with improved efficacy and safety. Notably the panel identified reduction in caregiver burden and reducing utilization of surgery as potential benefits of novel therapies. Molecular subtypes of DDLPS are promising therapeutic targets of novel therapies. Treatments inhibiting CDK4/6 and MDM2 have demonstrated benefit in early trials among patients with DDLPS that have chromosomal amplification of these genes [32]. In addition, immune checkpoint blockers have been evaluated alone and as part of combination regimens and with promising clinical activity in some tumor subtypes [32,33,34,35].

The consensus statements generated from this study contribute to our understanding of DDLPS management in the US, and address evidence gaps for a rare disease with little published information. There have been limited advancements in medical treatment for DDLPS since doxorubicin became the standard first-line treatment for metastatic soft tissue sarcomas over 3 decades ago [8]. Recent evidence shows that doxorubicin-based regimens remain the most common systemic treatment for DDLPS [36]. Experts strongly agreed on the need for a new systemic treatment that offers substantial improvement in efficacy over doxorubicin. New treatments currently in clinical trials offer hope for better outcomes and have the potential to disrupt current treatment algorithms. The panel highlighted the specific need for the development of new therapies that reduce the average number of surgeries patients receive which is a significant source of patient and caregiver burden.

A few limitations should be considered in the results of this Delphi study. First, there are general limitations to the Delphi study process including a lack of standard criteria to define consensus and the opportunity to introduce bias through panelist selection and Survey 1 construction. The Delphi panel was limited to 9 medical and surgical oncologists with expertise in DDLPS allowing for all perspectives to be captured during the virtual workshop. However, in limiting the panel to this size the opinions and consensus statements may not fully reflect practice norms across the United States, particularly at centers that treat few patients with DDLPS. Focusing the study on the diagnosis, treatment, and unmet needs of DDLPS in the United States, also limited the generalizability of consensus statements to other geographies and excluded global perspectives. Lastly, while the aim of the study was to establish clinical expert consensus statements, the study did not capture patient perspectives on the consensus statements. The absence of patient perspectives in the study may limit the consensus statements to accurately and fully reflect the burden of disease or treatment preferences among key stakeholders. Surveys or interviews with DDLPS patients are needed to better understand the real-world experiences in the diagnosis, treatment, and remaining unmet needs.

## 5. Conclusions

The first Delphi panel of clinical experts in the United States generated areas of consensus for the unmet need for the diagnosis and treatment of DDLPS. These areas improve the understanding of decision factors for surgical and non-surgical treatments and anticipated utilization of novel therapies.

## Figures and Tables

**Table 1:** Delphi panel participants.

Participant Name	Affiliation
Bruce Brockstein, MD	Kellogg Cancer Center, Endeavor Health; Evanston, IL
Andrew Fang, MD	Kaiser South San Francisco Medical Center; San Francisco, CA
Alex Haynes, MD, MPH, FACS	University of Texas; Austin, TX
Gregory Litton, MD	Intermountain Medical Center, Murray, UT
Minggui Pan, MD, PhD	Stanford University; Stanford, CA
Parth Shah, MD	Saint Joseph Hospital, Intermountain Health; Denver, CO
Shiraj Sen, MD, PhD	Next Oncology, San Antonio, TX
Jonathan C. Trent, MD, PhD	Sylvester Comprehensive Cancer Center, University of Miami Health System; Miami, FL
Shawn Young, MD	Saint Joseph Hospital, SurgOne; Denver, CO

**Table 2:** Diagnosis of DDLPS consensus statements.

Statement	Statement	Agreement Achieved
1	The likelihood a patient with DDLPS will present with symptoms at the time of diagnosis varies by primary tumor location.	Round 1
2	Many disciplines including surgical oncology, orthopedic oncology, radiology, interventional radiology, and pathology are typically consulted in the pathway to diagnose DDLPS.	Round 2
3	Patients on average have at least 2–3 referrals before a DDLPS diagnosis is made.	Round 2
4	An accurate DDLPS diagnosis is typically made within a year from symptom onset.	Round 2
5	Common reasons affecting the timely identification, and diagnosis of DDLPS patients include non-specific symptoms, delays in receiving biopsy or imaging, and clinician misdiagnosis.	Consensus Meeting

**Table 3:** Treatment of DDLPS consensus statements.

Statement	Statement	Agreement Achieved
6	Surgery with or without radiation or chemotherapy is the preferred first treatment in early-stage disease, in patients diagnosed with DDLPS.	Round 2
7	Once diagnosed, approximately 80% of stage 3 DDLPS patients undergo an oncologic surgery as part of a treatment strategy with curative intent.	Round 2
8	For patients with stage 3 DDLPS, surgical morbidity, tumor location, expected adequacy of margins, and comorbidities are the most influential factors in determining if a patient should not receive surgery as first-line of treatment.	Consensus Meeting
9	For patients initially diagnosed as stage 3 for whom surgery is not recommended, approximately 80% will receive systemic therapy.	Consensus Meeting
10	Among patients that receive surgery as initial treatment and have disease progression, tumor location, resectability, symptom burden, performance status, time interval since surgery, and presence of metastases are the most important factors for determination of next treatment selection.	Round 1
11	A DDLPS patient should be recommended for systemic therapy, versus an additional surgery, if disease progressed distantly within 6 months of surgery.	Consensus Meeting
12	For patients initially diagnosed as stage 3 who progress after surgery, approximately 80% will receive systemic therapy.	Consensus Meeting
13	An estimated 30% of stage 4 DDLPS patients receive palliative surgery with utilization dependent on primary tumor location.	Consensus Meeting
14	An estimated 10% of patients with stage 4 DDLPS will never receive systemic therapy.	Consensus Meeting
15	Doxorubicin-based chemotherapy is the preferred first treatment typically provided to patients diagnosed with unresectable or metastatic DDLPS.	Round 1
16	Doxorubicin or doxorubicin-based chemotherapy is the most commonly used first-line systemic treatment for DDLPS.	Round 2
17	Ifosfamide, gemcitabine with or without docetaxel, pazopanib, trabectedin, eribulin, palbociclib are the most commonly used treatment regimens for subsequent line treatment of DDLPS (after doxorubicin).	Consensus Meeting

**Table 4:** DDLPS unmet need consensus statements.

Statement	Statement	Agreement Achieved
18	For patients with stage 3 or 4 DDLPS, the greatest unmet need is for more efficacious systemic therapies.	Round 2
19	There is a need for a first-line treatment with improved efficacy in the treatment of locally advanced (unresectable) and metastatic DDLPS.	Round 1
20	There is a need for a first-line systemic treatment with improved safety and tolerability in the treatment of locally advanced (unresectable) or metastatic DDLPS.	Round 1
21	For patients with unresectable DDLPS, the greatest need is for a new systemic treatment that improves efficacy that can be used before other currently available systemic therapies.	Round 2
22	Locally advanced (unresectable) or metastatic DDLPS limits patient and caregivers’ daily personal and/or professional life.	Round 1
23	Currently among patients that receive surgery for DDLPS, the average number of surgeries patients have prior to systemic therapy is 1–4.	Round 2
24	The availability of a new systemic treatment that offers substantial improvement in efficacy over doxorubicin has the potential to reduce the average number of surgeries.	Round 2
25	A new systemic treatment for DDLPS which reduces the need for additional surgeries would meaningfully reduce patient and caregiver burden.	Round 1

## Data Availability

The original contributions presented in this study are included in the article and Supplementary Materials. Further inquiries can be directed to the corresponding author.

## Supplementary Materials

The following supporting information can be downloaded at: https://www.mdpi.com/article/10.3390/cancers17172815/s1, The full list of consensus and non-consensus statements from Survey 1, Survey 2, and virtual workshop.

## Abbreviations

The following abbreviations are used in this manuscript:

CDK4	Cyclin-dependent kinase 4
DDLPS	Dedifferentiated liposarcoma
MDM2	Mouse double minute 2
WDLPS	Well-differentiated liposarcoma
