# Supplementary material for: Diagnosis, Treatment, and Unmet Needs of Dedifferentiated Liposarcoma in the United States: A Multidisciplinary Delphi Study
Source: Cancers (Basel). 2025 Aug 28;17(17):2815. doi: 10.3390/cancers17172815 (PMC12427463; doi:10.3390/cancers17172815)
Supplement: Supplementary file 1 [file cancers-17-02815-s001.zip › cancers-3772789-supplementary.pdf]

Diagnosis, Treatment, and Unmet Needs of Dedifferentiated Liposarcoma in the United States: a Multidisciplinary Delphi Study

Supplemental Material

Survey 1 Results

Diagnosis of DDLPS

1. In which practice setting do you mostly work or provide treatment?
   a) Community institution - 6
   b) Specialty/academic institution - 2
   c) Other (please describe) - 0

2. What disciplines (e.g. pathology, radiology, surgery, etc.) are typically consulted, and diagnostic tools and/or procedures are used, in the pathway to diagnose DDLPS?
   - Surgical Oncology/Orthopedic Oncology, Radiology, Pathology (in that order). Sometimes Medical Oncology will be involved. Often a General Surgeon or Primary Care Provider is the initiator of referral. Other sources could be urology (retroperitoneal tumors interpreted as being of renal origin) or gynecology (pelvic tumors in women interpreted as being of ovarian origin)
   - Disciplines: Pathology, Radiology, Nuclear Medicine, Surgical Oncology, Radiation Oncology, Medical Oncology. Biopsy performed typically via percutaneous or incisional biopsy. Biopsy may be performed by interventional radiology or surgeon.
   - Surgical Oncology or Orthopedic Oncology, Pathology with routine and immunohistochemical studies and NGS studies if needed, radiology, radiation Oncology
   - Pathology, surgical oncology, orthopedic oncology, radiation oncology, PET/CT, MRI, and CT scans are used.
   - Radiology (PET/CT scan or CT chest, abdomen, and pelvis with optional MRI based on the case), interventional radiology (for biopsy), pathology (for staining and molecular testing needed to make the diagnosis), and medical oncology to review all of the above.
   - Pathology, radiology, surgical oncology, medical oncology, radiation oncology, supportive services (genetic counseling, nutrition, psychosocial, etc.)

- Surgical oncology, radiation oncology, medical oncology, radiology, interventional radiology, pathology, genetics, urology, vascular surgery
- Radiology and pathology. Pathology typically including morphology and IHC and NGS

3. By your estimation, what proportion of patients present with symptoms at the time of diagnosis?

a) 5%
b) 10%
c) 20
d) 30%
e) 40% - 2
f) 50% - 1
g) 60% - 1
h) 70% - 1
i) 80% - 1
j) 90% - 2
k) 95%
l) 100%

4. The likelihood a patient with DDLPS will present with symptoms at the time of diagnosis varies by primary tumor location.

a) Agree (7-9) - 7
b) Neither agree nor disagree (4-6) - 0
c) Disagree (1-3) - 1

5. By your estimation, patients on average have how many referrals before a DDLPS diagnosis is made?

a) 1 - 1
b) 2 - 6
c) 3 - 1
d) 4+

6. Are you typically the person making the DDLPS diagnosis and if patients are referred to you, from what practice/specialty are they usually referred from?
   - Yes. PCP or general surgeon most commonly. Also, urology or gynecology.
   - Yes. I will typically ensure that patients have appropriate imaging including an MRI with contrast or CT scan with contrast. Based on imaging an IR biopsy, in-office tru-cut biopsy, or incisional biopsy will be recommended.
   - patients are usually referred to me from a surgical oncologist, occasionally from a primary care div provider who has obtained a biopsy via radiologic CT biopsy and then referred as they know I have a subspecialty sarcoma practice
   - typically, patient is referred with a diagnosis already by primary physician or surgeon.
   - Often from a surgeon if not directly referred to me by a PCP or ER/hospital
   - 50/50. In the retroperitoneum, these patients are most commonly referred to surgical oncology without a biopsy performed. Depending on size entirely (< 3cm), these patients with extremity lesions may undergo excisional biopsy by the general surgeon. If larger, most of these patients are referred based on imaging alone prior to biopsy.
   - Majority of the times yes, I am the person making the diagnosis. Usually, the patient is referred to me by medical oncology or general surgery.
   - I'm a medical oncologist so I do not make pathological diagnoses. Patients are most commonly referred to me from surgeons that sometimes from primary care doctors who have made a diagnosis

7. The average timeline to an accurate DDLPS diagnosis is (please select one)

a) 0-3 months - 6
b) 4-6 months - 1
c) 7-12months - 1
d) Between 1-2 years
e) Between 2-3 years
f) More than 3 years
g) Other (please describe below)

8. Timely identification, and diagnosis of DDLPS patients is a challenge in your healthcare setting.

a) Agree (7-9) - 2

b) Neither agree nor disagree (4-6) - 4
c) Disagree (1-3) - 2

9. There are gaps in the diagnosis of DDLPS during the pre-symptomatic or pre-surgery stages of the disease based on site of care (community vs academic or specialty center)

a) Agree (7-9) - 1
b) Neither agree nor disagree (4-6) - 4
c) Disagree (1-3) - 3

Treatment of DDLPS

10. Surgery is the preferred first treatment in early state disease, in patients diagnosed with DDLPS

a) Agree (7-9) - 6
b) Neither agree nor disagree (4-6) - 2
c) Disagree (1-3) - 0

11. Doxorubicin-based chemotherapy is the preferred first treatment (medication or procedure) typically provided to patients diagnosed with unresectable or metastatic DDLPS

a) Agree (7-9) - 8
b) Neither agree nor disagree (4-6) - 0
c) Disagree (1-3) - 0

12. Among newly diagnosed patients with stage 3 or 4 DDLPS, please estimate the proportion of patients that receive each of the following as a first line treatment (must sum to 100%):

a) Definitive radiotherapy – 9%
b) Systemic therapy – 45%
c) Surgery – 41%
d) Best supportive care – 3%
e) Observation / No treatment – 2%

13. By your estimation, what proportion of stage 3 DDLPS patients are eligible for surgery?

a) 5%
b) 10%
c) 20%
d) 30%
e) 40%
f) 50% - 1
g) 60% - 1
h) 70% - 1
i) 80% - 1
j) 90% - 3
k) 95% - 1
l) 100%

14. By your estimation, what proportion of stage 4 DDLPS patients are eligible for surgery?

a) 5% - 2
b) 10% - 1
c) 20% - 1
d) 30% - 2
e) 40%
f) 50% - 2
g) 60%
h) 70%
i) 80%
j) 90%
k) 95%
l) 100%

15. Among patients that receive surgery as initial treatment and have disease progression, the factors that are most important for determination of next treatment selection include:

1. Age
    a. Agree (7-9) - 3
    b. Neither agree nor disagree (4-6) - 4

   c. Disagree (1-3) - 1
2. Performance status
   a. Agree (7-9) - 8
   b. Neither agree nor disagree (4-6) - 0
   c. Disagree (1-3) - 0
3. Symptom burden
   a. Agree (7-9) - 7
   b. Neither agree nor disagree (4-6) - 1
   c. Disagree (1-3) - 0
4. Tumor resectability
   a. Agree (7-9) - 8
   b. Neither agree nor disagree (4-6) - 0
   c. Disagree (1-3) - 0
5. Tumor location
   a. Agree (7-9) - 7
   b. Neither agree nor disagree (4-6) - 1
   c. Disagree (1-3) - 0
6. Tumor grade and size
   a. Agree (7-9) - 4
   b. Neither agree nor disagree (4-6) - 4
   c. Disagree (1-3) - 0
7. Presence of metastases
   a. Agree (7-9) - 7
   b. Neither agree nor disagree (4-6) - 1
   c. Disagree (1-3) - 0
8. Quality of surgical margins
   a. Agree (7-9) - 4
   b. Neither agree nor disagree (4-6) - 3
   c. Disagree (1-3) - 1
9. Preoperative/intraoperative tumor rupture
   a. Agree (7-9) - 4
   b. Neither agree nor disagree (4-6) - 3
   c. Disagree (1-3) - 1
10. Time since prior surgery
   a. Agree (7-9) - 6
   b. Neither agree nor disagree (4-6) - 1
   c. Disagree (1-3) - 1

16. The threshold for specific factors where a patient would be recommended for systemic therapy versus an additional surgery include: (please answer for each criterion, or write “threshold cannot be defined” where you cannot provide a specific value)

5. Age greater or equal to…
    a. Threshold cannot be defined - 5
    b. 80
    c. Life expectancy less than 5 years
6. Performance status less than or equal to…
    a. Threshold cannot be defined - 4
    b. 2 - 2
    c. 3 - 2
7. Symptom burden described as…
    a. Clear symptoms related to tumor
    b. Extensive mets will bias toward systemic therapy
    c. Persistent and progressing symptoms affecting mobility quality of life not amenable to surgery palliation
    d. How acute and severe
    e. N/A because this doesn’t drive decision making
    f. Unlikely to be resolved for more than 1-2 months.
    g. Significant enough to affect quality of life
    h. Cannot resect all of the tumor recurrence or the asymptomatic part of the tumor recurrence
8. Tumor presence on…
    a. Anatomic location (visceral mets, encasement of neurovascular structures) biases towards systemic therapy
    b. Vital structures that are not resectable or resectable with significant surgical morbidity
    c. Visceral organs or peripheral lung or extremity
    d. A vital organ may make patient ineligible for surgery
    e. Threshold cannot be defined
    f. Imaging with major vascular/neural involvement
    g. A Structure that makes it unresectable
9. Tumor grade and size greater or equal to…
    a. Threshold cannot be defined – 3
    b. Anatomic location and resectability will be more important than tumor size or grade for surgery

c. Lower grade tumors or tumors with mixed low-grade high-grade features with the low-grade histology predominating may benefit more from surgery.
d. Tumor is always grade 2 or 3 and would not matter, size is not as important, it is the location and resectability that matters.
e. N/A because this is important moreso based on if it's impinging on another organ or not
f. grade 3, size threshold cannot be defined, location important

10. Presence of metastases... (anywhere or specify locations of concern)
    a. Widespread visceral and an expected life span of less than 6 months may make nonoperative care more likely
    b. Widespread metastases that are bulky generally would receive systemic chemotherapy
    c. Presence of metastasis would typically indicate surgery is not the best option.
    d. Would only operate if patient has oligometastatic disease
    e. The vast majority of these patients would not be offered surgery outside of a clinical trial or symptom management/mitigate
    f. Distant metastases outside the regional basin
    g. Multiple metastases precluding resection or where a predominant tumor volume could not be removed
11. Surgical margins are...
    a. Threshold cannot be defined - 3
    b. If unresectable, then systemic therapy is likely recommended
    c. Positive for likely to be positive with additional resection would favor chemotherapy
    d. If it is going to be a R2 resection then surgery would not make sense
    e. Predictably going to be negative when we operate; sometimes we have to leave a positive margin on the IVC and try to graft this later; gynecology sometimes come to do a hysterectomy too
    f. Grossly positive
12. Time since prior surgery is...
    a. Threshold cannot be defined - 1
    b. Short
    c. Rapid recurrence, less than 3-6 months will push towards systemic therapy
    d. Less than 6 months would favor chemotherapy unless margins were close and additional margins can be obtained without significant surgical morbidity
    e. If it is within a few months then that would make is less desirable.

f. >6 months makes us more inclined to operate
g. Time less than 6 months likely would not benefit from additional surgery.
h. Generally less than 12 months

17. The most commonly used first line systemic treatment regimens for DDLPS are:

1. 1st most used treatment
    a. Adriamycin / Doxorubicin - 8
2. 2nd most used treatment
    a. Adriamycin/Ifosfamide – 3
    b. Gemzar/docetaxel – 2
    c. Ifosfamide – 2
    d. Doxorubicin plus ifosfamide - 1
    e. Votrient (pazopanib) - 1
3. 3rd most used treatment
    a. Trabectedin
    b. Palbociclib or pazopanib
    c. Gemcitabine
    d. Combination chemotherapy, etc.
    e. Gemcitabine single agent

18. Please select the option that best describes your use of ifosfamide for first line systemic treatment of DDLPS:

a) Never - 1
b) Rarely - 1
c) Sometimes - 6
d) Often
e) Always

19. The most commonly used treatment regimens for subsequent line treatment of DDLPS are:

1. 1st most used treatment
    a. Doxorubicin, Votrient (Pazopanib), Gemzar/docetaxel, Gemcitabine plus Taxotere - 2, Ifosfamide – 2,
2. 2nd most used treatment

    a. Eribulin, trabectedin, pazopanib, Gemcitabine monotherapy, Clinical trial consideration, PD-1 inhibitor
3. 3rd most used treatment
    a. Trabectedin - 2, Clinical trial, Palbociclib, CDK 4/6 inhibitor

Unmet Need

20. For patients with stage 3 or 4 DDLPS, the greatest unmet need is (please select one):

a) Poor efficacy of available systemic therapies - 8
b) Poor tolerability of available systemic therapies
c) Lack of systemic treatment options after progression
d) Other (please describe below)

21. I believe there is a need for a first line treatment with improved efficacy in the treatment of locally advanced (unresectable) or metastatic DDLPS

a) Agree (7-9) - 8
b) Neither agree nor disagree (4-6) - 0
c) Disagree (1-3) - 0

22. I believe there is a need for a first line systemic treatment with improved safety and tolerability in the treatment of locally advanced (unresectable) or metastatic DDLPS

a) Agree (7-9) - 8
b) Neither agree nor disagree (4-6) - 0
c) Disagree (1-3) - 0

23. Based on your clinical experience, what are the top 3 current diagnostic/clinical/medical unmet needs in locally advanced (unresectable) or metastatic DDLPS?

- Effective systemic therapy, Tolerated systemic therapy, Prognostic tools for treatment selection
- Effective systemic therapies that benefit a higher proportion of patients, Effective systemic therapies with better tolerance and safety profiles, Better targeted therapies (individualized or precision approaches) given the heterogeneity of tumors

- Preop multidisciplinary discussions and sarcoma-based conferences, Systemic therapy
- We need much more effective drugs, Diagnostic is a not an issue, More effective immunotherapy would be highly desirable
- Need for more efficacious therapies, Need for safer therapies, Need for a non-invasive way to diagnose DDLPS in minimally symptomatic patients whose diagnosis is delayed due to PCPs not getting imaging
- Efficacious systemic therapy, Widely available access to clinical trials, Quality of imaging to assess tumor margin, especially in the retroperitoneum
- Neoadjuvant chemotherapy, Targeted therapy, Reduced risk from radiation therapy
- Better therapeutics with lasting benefit, Better clarification of significance of the dedifferentiated component of a mixed tumor and the grade of that component, 2 versus 3, Prediction for when a local recurrence can be watched

24. Locally advanced (unresectable) or metastatic DDLPS limits patient and caregivers' daily personal and/or professional life

a) Agree (7-9) - 8
b) Neither agree nor disagree (4-6) - 0
c) Disagree (1-3) - 0

25. For patients with resectable DDLPS, there is greatest need for a new a systemic treatment that improves PFS:

a) Before surgery or currently available systemic therapies - 5
b) After surgery and before other systemic treatments - 5
c) After surgery and other systemic treatments - 2

26. For patients with unresectable DDLPS, there is greatest need for a new a systemic treatment that improves PFS:

a) Before other currently available systemic therapies - 8
b) After other currently available systemic treatments - 2

27. DDLPS patients receive too many additional surgeries due to a lack of efficacy with available systemic treatments

a) Agree (7-9) - 4
b) Neither agree nor disagree (4-6) - 3
c) Disagree (1-3) - 1

28. Currently, among patients that receive surgery for DDLPS the average number of surgeries patients have on average prior to systemic therapy is:

a) 1 - 2
b) 2 - 3
c) 3 - 2
d) 4 - 1
e) 5
f) 6
g) 7
h) 8+

29. With the availability of a new systemic treatment that nearly doubles mPFS vs doxorubicin with comparable safety and tolerability, I anticipate the average number of surgeries will be reduced.

a) Agree (7-9) - 5
b) Neither agree nor disagree (4-6) - 3
c) Disagree (1-3) - 0

30. A new systemic treatment for DDLPS which reduced the need for additional surgeries would meaningfully reduce patient and caregiver burden

a) Agree (7-9) - 8
b) Neither agree nor disagree (4-6) - 0
c) Disagree (1-3) - 0

Survey 2 Results

Diagnosis of DDLPS

1. Many disciplines including surgical oncology, orthopedic oncology, radiology, and pathology are typically consulted, in the pathway to diagnose DDLPS.
    a. Agree (7-9) – 8
    b. Neither agree nor disagree (4-6) – 0
    c. Disagree (1-3) – 0

2. Patients on average have at least 2-3 referrals before a DDLPS diagnosis is made.
    a. Agree (7-9) – 7
    b. Neither agree nor disagree (4-6) – 1
    c. Disagree (1-3) - 0

3. An accurate DDLPS diagnosis is typically made within a year from symptom onset.
    a. Agree (7-9) – 7
    b. Neither agree nor disagree (4-6) – 1
    c. Disagree (1-3) - 0

4. What gaps do you see for the timely identification, and diagnosis of DDLPS patients?
    - "Fear of biopsy" among non-specialist providers, leading to ill-conceived initial surgical treatment.
    - The abdominal symptoms are often not specific and providers will treat the symptoms for common non-malignant conditions which often led to delayed diagnosis. The symptoms resulted from an extremity mass are less likely to experience substantial delays as a palpable mass can cause more alarms for further workup. The gap remains provider recognition of disease and timely imaging studies.
    - I am not so sure how this could be solved as the symptoms are nonspecific and require a high level of suspicion, which often does not exist until well into the presentation.
    - The vast majority of soft tissues in the body are benign (lipomas, etc.). Given the rarity of DDLPS, recognition of an enlarging worrisome mass can be challenging. Symptoms may vary based on location and size of the tumor. In cases of small tumors, symptoms may be limited.
    - Enhanced attention to symptoms esp related to deep tumors (retroperitoneum, buttock, thigh) including good exam or early cross sectional imaging

- Delay in use of advanced imaging from PCP and use general surgeons who feel mass is benign lipomatous process and initially operate
- Health care systems and primary care practices are largely going to value based models where there is significant resistance to do imaging. This diagnosis usually follows radiographic identification
- Lack of symptoms until larger in size or involving specific organs, lack of understanding among the general medical community/primary care physicians about the best imaging modality for early identification and time taken for referral to a surgeon/oncologist.

Treatment of DDLPS

5. Surgery with or without neoadjuvant radiation or chemotherapy is the preferred first treatment in early state disease, in patients diagnosed with DDLPS
   a. Agree (7-9) – 7
   b. Neither agree nor disagree (4-6) – 0
   c. Disagree (1-3) - 1

6. Approximately 80% of stage 3 DDLPS patients are eligible for surgery.
   a. Agree (7-9) – 8
   b. Neither agree nor disagree (4-6) – 0
   c. Disagree (1-3) - 0

7. For patients with stage 3 DDLPS, what factors are most influential in determining if a patient should surgery over systemic therapy?
   - Location of tumor and morbidity of resection. Anticipated surgical margins. Comorbid disease making either surgery or systemic therapy less appealing
   - for abdominal DDLPS, the relationship of the tumor with the adjacent structures and the skills of available surgeons and the experience of the treating oncologist tend to be determining factors of patient's treatment approach.
   - Most of these patients are candidates for surgery as the preferred first treatment. One might consider systemic therapy first if concerns for potential metastatic disease exists and a period of observation may clarify. Additionally, anticipated postoperative R status is paramount. If one does not believe and R0 result is likely, preoperative chemotherapy may be indicated. Finally, patient comorbidities preventing aggressive surgical treatment might favor chemotherapy as a feasible option.
   - Location, size and resectability for adequate margins.

- Ability to achieve a R0 or at least R1 resection. (tumor and location characteristics). Medical operability/ comorbidities
- Ability for surgeon to obtain adequate margins without excessive surgical morbidity and absence of metastatic or multifocal disease
- Overall health and performance status (ie clearance/safety for surgery), size, location, patient preference
- 1) Location 2) Involvement of surrounding structures 3) Existing morbidity from the tumor vs the morbidities expected following surgery 4) Patient co-morbidities 5) Toxicities from systemic therapy

8. For patients with stage 3 DDLPS, what proportion of patients will never receive systemic therapy?
    a. 5% - 1
    b. 10%
    c. 20%
    d. 30% - 3
    e. 40% - 1
    f. 50% - 1
    g. 60% - 1
    h. 70% - 1
    i. 80%
    j. 90%
    k. 95%
    l. 100%

9. By your estimation, what proportion of stage 4 DDLPS patients are eligible for palliative surgery?
    a. 5%
    b. 10%
    c. 20% - 3
    d. 30% - 2
    e. 40%
    f. 50% - 2
    g. 60% - 1
    h. 70%
    i. 80%
    j. 90%
    k. 95%

l. 100%

10. For patients with stage 4 DDLPS, what proportion of patients will never receive systemic therapy?
    a. 5% - 1
    b. 10% - 3
    c. 20% - 2
    d. 30% - 1
    e. 40% - 1
    f. 50%
    g. 60%
    h. 70%
    i. 80%
    j. 90%
    k. 95%
    l. 100%

11. A DDLPS patient should be recommended for systemic therapy versus an additional surgery if disease progressed within 6 months of the surgery.
    a. Agree (7-9) – 6
    b. Neither agree nor disagree (4-6) – 2
    c. Disagree (1-3) - 0

12. Doxorubicin is the most commonly used first line systemic treatment regimens for DDLPS
    a. Agree (7-9) – 8
    b. Neither agree nor disagree (4-6) – 0
    c. Disagree (1-3) - 0

13. Ifosfamide, gemcitabine with docetaxel, and pazopanib are the most commonly used treatment regimens for subsequent line treatment of DDLPS.
    a. Agree (7-9) – 6
    b. Neither agree nor disagree (4-6) – 2
    c. Disagree (1-3) - 0

DDLPS Unmet Need

14. For patients with stage 3 or 4 DDLPS, the greatest unmet need is poor efficacy of available systemic therapies

    a. Agree (7-9) – 8
    b. Neither agree nor disagree (4-6) – 0
    c. Disagree (1-3) - 0

15. For patients with unresectable DDLPS, there is greatest need for a new a systemic treatment that improves efficacy is before other currently available systemic therapies
    a. Agree (7-9) – 7
    b. Neither agree nor disagree (4-6) – 0
    c. Disagree (1-3) - 0

16. Which of the following treatment options might be appropriate if a new systemic treatment option with proven efficacy in the unresectable setting becomes available?
    a. Adjuvant use - 6
    b. Neoadjuvant use - 7
    c. Treatment of patients with well differentiated LPS - 3

17. Currently among patients that receive surgery for DDLPS, the average number of surgeries patients have on prior to systemic therapy is 1-4
    a. Agree (7-9) – 7
    b. Neither agree nor disagree (4-6) – 1
    c. Disagree (1-3) - 0

18. The availability of a new systemic treatment that offers substantial improvement in efficacy over doxorubicin has the potential to reduce the average number of surgeries.
    a. Agree (7-9) – 8
    b. Neither agree nor disagree (4-6) – 0
    c. Disagree (1-3) - 0
